# Incidence of maternal *Toxoplasma *infections in pregnancy in Upper Austria, 2000-2007

**DOI:** 10.1186/1471-2334-11-348

**Published:** 2011-12-14

**Authors:** Ulrich Sagel, Alexander Krämer, Rafael T Mikolajczyk

**Affiliations:** 1 Department of Public Health Medicine, School of Public Health, University of Bielefeld, P.O. Box 10 01 31, D-33501 Bielefeld, Germany; 2 analyse BioLab GmbH, Eisenhandstr. 4-6, A-4020 Linz, Austria; 3 Institute of Hygiene and Mikrobiology, Lower Austria State Hospital of St. Pölten-Lilienfeld, Probst-Führer-Str. 4, A-3100 St. Pölten, Austria; 4 Bremen Institute for Prevention Research and Social Medicine, Achterstr. 30, D-28359 Bremen, Germany

## Abstract

**Background:**

Despite three decades of prenatal screening program for toxoplasmosis in Austria, population-based estimates for the incidence of maternal infections with *Toxoplasma gondii *during pregnancy are lacking. We studied the incidence of primary maternal infections during pregnancy in the Federal State of Upper Austria.

**Methods:**

Screening tests for 63,416 women and over 90,000 pregnancies (more than 84.5% of pregnancies in the studied region) in the time period between 01.01.2000 and 31.12.2007 were analysed. The incidence of toxoplasmosis was estimated indirectly by binomial and directly by interval censored regression.

**Results:**

During the studied period, 66 acute infections (risk of 0.07% per pregnancy) were detected, but only 29.8% of seronegative women were tested at least three times during their pregnancies. The seroprevalence of *Toxoplasma *antibodies among all tested women was 31%. Indirectly estimated incidence (from differences in prevalence by age) was 0.5% per pregnancy, while directly estimated incidence (interval censored regression) was 0.17% per pregnancy (95% confidence interval: 0.13-0.21%).

**Conclusions:**

Calculating incidence from observed infections results in severe underreporting due to many missed tests and potential diagnostic problems. Using statistical modelling, we estimated primary toxoplasmosis to occur in 0.17% (0.13-0.21%) of all pregnancies in Upper Austria.

## Background

Congenital toxoplasmosis is among the infections associated with a high risk of complications, but fortunately acute infections during pregnancy are relatively rare [1,2]. Due to the potential to cause life-long disability, the burden of disease of congenital toxoplasmosis is considerable [3]. In order to prevent foetal infections and complications of toxoplasmosis, screening programs during pregnancy and a subsequent treatment of identified maternal primoinfections were introduced in a few countries [1,2,4-6].

Austria was the first country to start with population-wide free screening and treatment of maternal infections in 1975, soon followed by France. Nonetheless, little is known about the incidence of these infections from these countries despite of their long tradition of toxoplasmosis prevention [7]. We used data from a screening laboratory that covers most of the population of one federal state in Austria in an attempt to determine the incidence in this region.

## Methods

### Sample

We retrospectively analysed serological data of all pregnant women aged 15-45 years insured by the OÖGKK ("Oberösterreichische Gebietskrankenkasse": Upper Austrian Regional Health Insurance) and place of residence in Upper Austria. The OÖGKK is the largest statutory health insurance company in Upper Austria. Based on a special agreement with the health insurance company, all serological tests for *Toxoplasma*-specific IgG and IgM antibodies were conducted in one single laboratory (analyse BioLab GmbH, Linz). Information on gestational week when the screening was performed and the date of delivery was not available. We included only women for whom it could be assumed that their last test in a given pregnancy was conducted in the period from 01.01.2000 to 31.12.2007. Tests were classified as belonging to the same pregnancy when they were performed within a time window of 200 days (the analysis was also repeated using 300 days as a time window). According to the regulations in Austria, screening has to be performed before the sixteenth week of gestation and repeated in seronegative women in the fifth and eighth pregnancy month [4]. Austrian experts recommended the application of shorter, eight-week screening intervals in 2005 [8].

### Diagnostic tools

**Figure 1:**
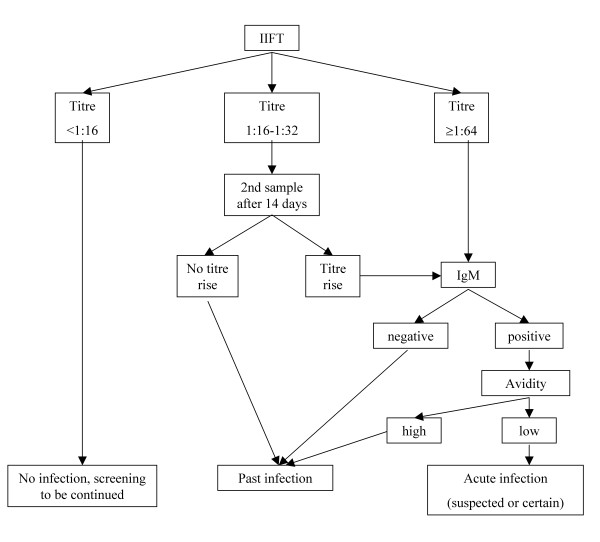
**Decision algorithm for classification of infections**.

The diagnostic algorithm is presented in Figure 1. Before October 2004, coated slides for IIFT were provided by the Clinical Institute of Hygiene and Medical Microbiology of the Medical University of Vienna, Division of Medical Parasitology and used with FITC-marked anti-human-IgG/A/M/D/E-conjugate from DiaSorin S.p.A., Saluggia, Italy. Since October 2004, the IIFT slides were replaced by a commercially available IIFT kit (bioMérieux, Marcy-l'Etoile, France). IgM-test (VIDAS TOXO IgM, bioMérieux) and IgG-avidity-test (VIDAS TOXO IgG AVIDITY, bioMérieux) were performed in cases of a positive IIFT test to rule out an acute infection.

All tests with an IIFT titer of 1:16 or higher were defined as seropositive. A suspected acute infection in pregnancy was defined by the following findings: anti-*Toxoplasma*-specific IgM-antibodies positive (>0.65) and low (<0.2) *Toxoplasma*-specific IgG-avidity. A suspected infection was considered as proven (and classified as certain infection in our analysis) when there was a more than fourfold antibody-titre rise. Given the difficulties of assessing the threshold in the IIFT when seroconversions occurred in a short time period, but were not accompanied by a positive IgM or a low avidity they were considered false positive and were excluded.

### Data flow and data protection

Data was extracted from the laboratory software BasuLab (Berger Analysen und Informationstechnik GmbH, Puchenau, Austria) and imported into STATA, version 8.2 (Statacorp, College Station, TX, USA) for all subsequent analyses (STATA-log-file available from the corresponding author on request). To ensure data protection and to meet the obligations of the Austrian data protection law (§ 46 (1) 2 and § 46 (5) Datenschutzgesetz 2000), personal identifiers were replaced by unique pseudonyms. Furthermore, the place of residence and its postal code were replaced by the corresponding NUTS-3 regions (AT311: "Innviertel", AT312: "Linz-Wels", AT313: "Muehlviertel", AT314: "Steyr-Kirchdorf", AT315: "Traunviertel" [9]) and an indicator variable for the three big cities of Linz, Wels or Steyr (the former two being part of region AT312 and the latter part of AT314). The study was reviewed and approved by the ethics committee of the Elisabethinen Hospital Linz, Austria.

### Statistical analysis

Firstly, we estimated the crude incidence from observed primoinfections during pregnancy. As testing did not cover the whole pregnancy for many seronegative women, we expected to miss a lot of infections and to underestimate the incidence. We therefore used further indirect and direct methods to estimate the true infection rate in pregnancy. From a binomial regression model, we estimated the increase in the seroprevalence per year of age and calculated the increase corresponding to the pregnancy duration of 268 days to obtain incidence under the assumption that differences in prevalence by age reflect new infections (indirect method) [10]. Since diagnosing seroprevalence is less error prone than correctly assessing the very rare event of acute infection, this method was robust against diagnostic errors. We subsequently analysed the incidence of *Toxoplasma *infections during pregnancy in seronegative women by means of interval censored regression (direct method) [11]. Interval censored regression allows one to account for the fact that in the case of a positive test it was only known that the infection occurred in the preceding time interval since the last negative test. Again, the estimate was recalculated to the period of 268 days. In order to obtain the incidence in relation to all pregnant women (as typically reported in other studies), the result was multiplied by (1-seropositive fraction). Since this analysis was based only on time during pregnancy, we were able to use information about IgM and avidity to rule out false positive results of the IIFT test. Within a pregnancy, screening tests were usually only about 3 or 4 months apart, and IgM remains positive and avidity low in this time span after an acute infection [12].

## Results

### Seroprevalence of *Toxoplasma *infections among pregnant women

**Figure 2:**
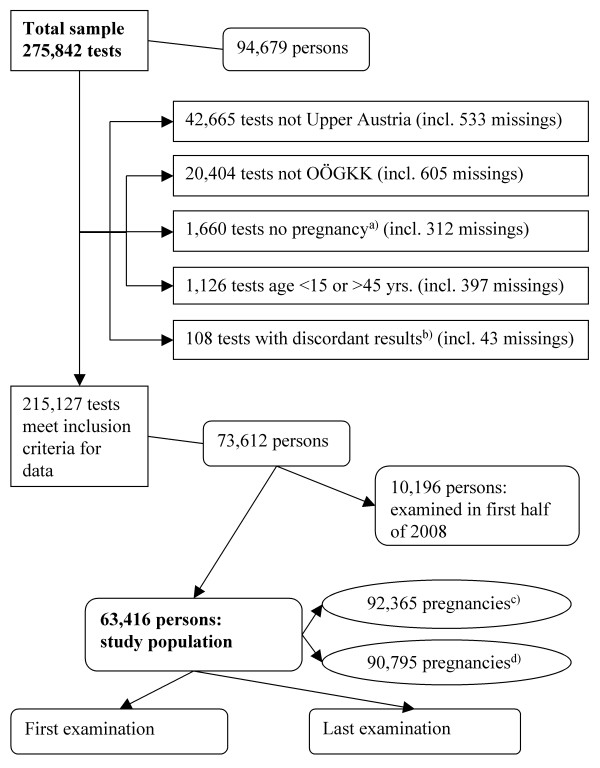
**Selection of study population a) few tests were ordered for not-pregnant persons, b) i.e., test results did not match the interpretation, or result or interpretation were missing on the report, c) 200-day estimate (see methods section), d) 300-day estimate (see methods section)**.

There were 275,842 test results in the database in total (Figure 2). Inclusion criteria for the study population were met by 63,416 women in the dataset. These women contributed 92,365 pregnancies, based on the 200 days estimate. This number only slightly decreased when a more conservative estimate of 300 days was used. The total population for the studied region is around 1.4 million. In Upper Austria, there were 109,327 life births in total in the years 2000-2007 [13]. The total number of pregnancies including spontaneous and induced abortion and stillbirths was certainly substantially higher, but most of the spontaneous and induced abortions will happen before the seroprevalence testing, leaving only the stillbirths (<0.1% of life births) which are unaccounted for. Dividing 92,365 pregnancies included in our study by 109,327 life births in the region, we concluded that our data covered more than 84.5% of all life births in Upper Austria during this period.

**Figure 3:**
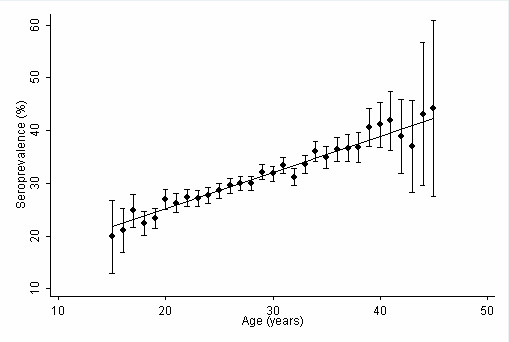
**Seroprevalence of *Toxoplasma *infections by age Seroprevalence according to first examination within study period**. The trend line is weighted by the underlying number of observations. Capped spikes: 95% confidence intervals.

At their first examination in the study period, women were in median 28.3 years old (interquartile range (IR) 24.3 to 32.2 years). The seroprevalence at the first examination was 30.6%. At their latest examination, women were on average 1.3 years older and the seroprevalence was slightly higher (31.7%). The seroprevalence increased in a linear manner with age (p < 0.01 for trend, Figure 3) and was significantly lower in cities (Table 1) than in the larger regions (p < 0.01, regardless of whether prevalence at first or latest examination was studied).

**Table 1:** Seroprevalence and observed (suspected and certain) primoinfections by region

Region^1)^	Prevalence (first)^2)^	Prevalence (last)^3)^	Suspected infections^4)^	Incidence suspected^5)^	Certain infections^6)^	Incidence certain^7)^	Pregn.^8)^	Inhab.^9)^
AT311	36.5	37.4	37	0.31	10	0.08	12,043	274,797

AT312	27.8	28.7	73	0.18	24	0.06	39,841	543,661

AT313	34.7	36.7	38	0.26	14	0.10	14,343	204,140

AT314	29.9	31.4	35	0.31	6	0.05	11,180	153,501

AT315	29.7	30.8	39	0.26	12	0.08	14,958	229,575

city^10)^	26.7	27.5	39	0.17	12	0.05	23,368	286,496

no city^11)^	32.0	33.2	183	0.27	54	0.08	68,997	1,119,178

Total	30.6	31.7	222	0.24	66	0.07	92,365	1,405,674

^1)^NUTS-3 regions [9] and the three biggest cities of Upper Austria

^2)^seroprevalence in percent at first examination

^3)^seroprevalence in percent at latest examination

^4)^number of suspected primoinfections

^5)^per 100 pregnancies (200-day estimate)

^6)^number of certain primoinfections

^7)^per 100 pregnancies (200-day estimate)

^8)^estimated number of pregnancies in study population (200-day estimate)

^9)^number of inhabitants in Upper Austria at 01.01.2007 [13]

^10)^i. e. Linz, Wels, Steyr

^11)^Upper Austria without Linz, Wels, Steyr

### Suspected and certain *Toxoplasma *primoinfections detected during pregnancy

The case definition for a suspected primoinfection during pregnancy was met by 222 women. Their median age was 27.8 years (IR 24.6-32.2 years). Of those 222 cases, 66 (29.7%) were classified as certain (i.e., these women had at least two tests with discordant results during the same pregnancy). Table 1 shows the distribution of the cases by regions and the corresponding incidence rates, with lower rates in cities than in regions including rural areas. The rural-urban difference was significant for suspected infections (p < 0.01), but not for certain infections (p = 0.18). The yearly numbers of cases ranged from 16 to 41 (mean: 26.5) for suspected infections and from 5 to 12 (mean: 8.3) for certain infections. No clear trend over time was observed (data not shown).

### Estimated incidence of acute *Toxoplasma *infections during pregnancy

The results of indirectly estimating incidence rates from age-related differences in seroprevalence are presented in Table 2 (first two columns). Consistent with estimates based on observed cases, the incidence rates appeared to be lower in the cities than in other regions, but the difference is not significant.

**Table 2:** Estimates of incidence of *Toxoplasma *infections during pregnancy (per 100 pregnancies), 95% confidence intervals in parentheses, separate models in each line

	Indirect estimate (Binomial regression model)		Direct estimate (Interval censored regression model)	
**Region/year^1)^**	**Incidence (first examination)^2)^**	**Incidence (latest examination)^2)^**	**Incidence^2)^**	**Incidence in seronegative women^2)^**

AT311	0.50 (0.37-0.63)	0.48 (0.35-0.62)	0.14 (0.07-0.30)	0.22 (0.11-0.47)

AT312	0.49 (0.43-0.56)	0.48 (0.41-0.55)	0.12 (0.08-0.19)	0.17 (0.11-0.27)

AT313	0.54 (0.41-0.68)	0.49 (0.35-0.63)	0.30 (0.18-0.47)	0.45 (0.28-0.73)

AT314	0.44 (0.31-0.58)	0.44 (0.31-0.58)	0.15 (0.07-0.32)	0.22 (0.10-0.46)

AT315	0.58 (0.47-0.70)	0.51 (0.39-0.63)	0.17 (0.10-0.31)	0.25 (0.14-0.45)

city^3)^	0.46 (0.37-0.55)	0.43 (0.34-0.52)	0.12 (0.07-0.22)	0.17 (0.09-0.30)

no city^4)^	0.52 (0.46-0.57)	0.49 (0.43-0.55)	0.18 (0.14-0.24)	0.26 (0.20-0.35)

Total	0.50 (0.46-0.55)	0.48 (0.43-0.52)	0.17 (0.13-0.21)	0.24 (0.18-0.31)

^1)^NUTS-3 regions [9] and the three biggest cities of Upper Austria

^2)^incidence is calculated per pregnancy, i.e. per 268-day interval

^3)^i. e. Linz, Wels, Steyr

^4)^Upper Austria without Linz, Wels, Steyr

The interval censored regression yielded a substantially lower estimate for incidence of toxoplasmosis in all pregnancies with 0.17% (0.13-0.21%) (Table 2, columns 3 and 4). The results were virtually unchanged when 300 days were used instead of 200 days to define tests belonging to one pregnancy. Similarly to the binomial regression model, the estimated incidence rates were slightly lower in the three biggest cities than in overall Upper Austria, but again the difference was statistically not significant. A model including calendar years did not show a significant change over time. Based on the findings from interval censored regression, we estimated that there were 152 (95% confidence interval: 118-196) acute *Toxoplasma *infections during pregnancy in the years 2000-2007 in the study sample (based on 92,365 pregnancies in the same period).

### Coverage of pregnancy with screening in seronegative women

In the study population, 38,576 women had their latest screening (based on the 200 days time window) and were seronegative in this examination. When only the latest pregnancy for each woman was included, we estimated that in 13.8% of those pregnancies one examination, in 56.5% two examinations, and in only 29.8% three or more examinations had been performed. From 2001 to 2007 the situation improved continuously, and in 2007, 35.5% of seronegative women were tested at least three times during pregnancy.

## Discussion

Our study estimated prevalence and incidence of toxoplasmosis and coverage with screening in pregnant women in Austria. The estimated seroprevalence of about 31% in pregnant women is in line with findings from other countries in Europe [7]. As expected, seroprevalence was higher in rural areas than in cities. The three recommended screening tests were conducted in only about 29.8% of seronegative women, despite the fact that about 95% of OÖGKK members attended all the check-ups of the Austrian maternal care program in pregnancy [14]. A recent study from a region in south-east France reported similar problems: Only 40% of pregnant women had all seven or more recommended tests [15]. Poor compliance to a complete screening program jeopardizes a direct analysis of the incidence of *Toxoplasma *infections in pregnancy. Consequently, incidence based on observed cases only resulted in severe underestimation if only certain diagnoses (0.07%) were considered. A certain diagnosis requires more than one test in pregnancy and, therefore, misses infections that occurred in early pregnancy before the first test. In addition, the period between the latest examination and birth is not included in the analysis. If only a single test result was available, infection could be only suspected, since high IgM and low avidity do not rule out a past infection [12,16]. Therefore, incidence based on observed suspected infections suffers from both an underestimation due to cases which were not observed, and an overestimation caused by false positive IgM and avidity tests.

Statistical methods are therefore necessary to derive estimates of true incidence. We used an indirect approach [10]: the age-specific seroprevalence suggested a linear association between age and seroprevalence (Figure 3), as also observed by others [7,10]. The estimates derived for incidence using this approach were higher than those obtained from observed suspected cases (0.5% per 100 pregnancies). While false test results are unlikely to cause a substantial overestimation in this method, differences in age-specific prevalence can be subject to age cohort effects, with a share of infections taking place in younger years of life but decreasing over time [17]. A decrease in the seroprevalence of *Toxoplasma *infections over time that may lead to overestimation in the indirect estimate has been observed in several European countries [10,18,19]. Consistently, a seroprevalence of 41% reported for 1995/96 in Upper Austria was considerably higher than our findings for 2000-2007 [20]. The reliability of the data for 1995/96 was questioned [21], but other reports from Austria also suggested a decreasing seroprevalence in the region, not only in humans but also in animals that are important for the transmission of disease to humans [22]. A decreasing trend is also in line with findings in The Netherlands comparing 1995/1996 and 2006/2007 [23]. Furthermore, the seroprevalence estimate is mostly based on the non-pregnant time. Women during pregnancy might be more conscious about avoiding potential sources of infection, such as eating undercooked meat and contact with contaminated soil [24]. Therefore, incidence of *Toxoplasma *infections during pregnancy in the same age group could be lower than in non-pregnant women. This effect might be partly compensated by an opposite bias, as pregnancy has been shown to be a risk factor for *Toxoplasma *infection in an epidemiological study from Brazil [25]. The authors assumed changes in lymphocyte functions during late pregnancy, which led to some level of immunosuppression towards protozoal infections and to explain this increased susceptibility. As late stages of pregnancy were underrepresented in our study due to the poor adherence to the screening scheme, changes in immunity might not play a major role. Overall, we conclude that estimating incidence from age-specific prevalence might not provide valid results for the true incidence.

The interval censored regression directly assessing incidence during pregnancy, appears to be the most appropriate approach to estimate the true incidence. However, the method is based directly on the rare event of acute infections and is therefore more affected by an imperfect specificity of testing. Interval censored regression depended on clear cut IIFT tests distinguishing seronegative from seropositive results and on IgM and avidity test results. We identified the following information regarding test characteristics: in the laboratory of analyse Biolab GmbH, 1,039 sera tested by IIFT were compared to the AxSYM and ARCHITECT test kits for anti-*Toxoplasma gondii*-IgG (Abbott Laboratories, Abbott Park, Illinois), with two investigators reading the IIFT. Sensitivity and specificity were 99.7% and 97.2% for the first investigator and 96.8% and 99.4% for the second investigator for AxSYM, and 99.7% and 98.3%/96.6% and 99.2% for ARCHITECT, respectively [Autengruber E, Linz 2008, unpublished bachelor's thesis]. According to the manufacturer's product information regarding sera from pregnant women, sensitivity of VIDAS IgM is 96.0% (95% confidence interval: 91.4-98.2%) and 100% of pregnant women with an acute infection not more than 4 months old show a low IgG antibody avidity (95% confidence interval: 98.1-100.0%). False positive results can be ruled out in the subsequent avidity testing, while false negative tests escape further diagnostics. Fortunately, sensitivity is particularly high, resulting in a marginal underestimation only. However, there is a potential mechanism which could cause a more substantial underestimation: using only times between tests during pregnancy excludes early pregnancy in which women might not be aware of being pregnant and thus be less careful in avoiding the exposure to toxoplasmosis. The contribution of this mechanism depends on the fraction of unplanned pregnancies and consciousness in avoiding sources of infection during early pregnancy.

### Strengths and limitations

The strength of our study is that we were able to analyse more than 84.5% of pregnancies leading to life births in Upper Austria. OÖGKK covers all social classes, the catchment area was clearly defined and only pregnant women were included. In most regions in Austria, screening is performed in several laboratories and it is difficult to assemble their screening data. Analysis of subsequent tests requires personal identifiers and exchange of this information between several institutes is complicated by personal data protection requirements. The use of routine data on toxoplasmosis testing in most other countries in the world (including the USA) is hampered by the fact that usually only privileged groups have access to screening.

Due to the missing information on parity, we could not provide separate estimates by parity. As seroprevalence increases with age, rates are also typically lower in primipara than in multipara. Unfortunately, we did not have any information about the gestational week at the time of infection. This information is important if complications of the infection should be studied. However, it is beyond the scope of this analysis to provide information about maternal-foetal transmission rates and the rate of children with clinical sequels in cases of congenital toxoplasmosis. Various studies gave heterogeneous information about these rates and were questioned with regard to their data quality [18,26].

We did not have information to study individual risk factors affecting incidence beyond place of residence. In an earlier analysis using the same data, a seasonal trend with a slight increase of diagnoses in winter (probably reflecting more infections in the fall) has been described [27,28].

Another problem is the clear allocation of patients to the study period. A pregnancy with several serological checks is not a time point but a time span. We used the last examination per pregnancy to decide on its allocation. In addition, we investigated a large, eight-year study period to reduce the number of pregnancies crossing the start or the end of the study period.

## Conclusions

Using statistical models, we estimated the incidence of maternal *Toxoplasma *primoinfections in pregnancy in Upper Austria, 2000 - 2007. All approaches to determine the incidence of *Toxoplasma *infections in pregnancy suffered from limitations. We consider the proportion of observed certain cases only (0.07%) the low bound and the estimate based on age-specific seroprevalence (0.5%) the high bound, and propose the interval censored regression model (0.17%) as the best estimate.

## Competing interests

The authors declare that they have no competing interests.

## Authors' contributions

All authors contributed to the design of the study and prepared and approved the final manuscript. US and AK conceived of the study. US prepared and anonymised the data for investigation. US and RTM performed the statistical analysis.

## Pre-publication history

The pre-publication history for this paper can be accessed here:

http://www.biomedcentral.com/1471-2334/11/348/prepub

## References

[B1] Remington JS McLeod R Thulliez P Desmonts G Remington JS, Klein JO, Wilson CB, Baker CJ Toxoplasmosis Infectious Diseases of the Fetus and the Newborn Infant 2006 6 Philadelphia: Elsevier Saunders 947 1091

[B2] Montoya JG Liesenfeld O Toxoplasmosis Lancet 2004 363 1965 1976 10.1016/S0140-6736(04)16412-X 15194258

[B3] Havelaar AH Kemmeren JM Kortbeek LM Disease burden of congenital Toxoplasmosis Clin Infect Dis 2007 44 1467 1474 10.1086/517511 17479945

[B4] Aspöck H Pollak A Prevention of prenatal toxoplasmosis by serological screening of pregnant women in Austria Scand J Infect Dis Suppl 1992 84 32 38 1290071

[B5] Thiébaut R Leproust S Chêne G Gilbert R the Systematic Review on Congenital Toxoplasmosis study group Effectiveness of prenatal treatment for congenital toxoplasmosis: a meta-analysis of individual patients' data Lancet 2007 369 115 122 17223474 10.1016/S0140-6736(07)60072-5

[B6] Bénard A Petersen E Salamon R Chêne G Gilbert R Salmi LR The European toxo prevention study group: survey of European programmes for the epidemiological surveillance of congenital toxoplasmosis Eurosurveill 2008 13 15 http://www.eurosurveillance.org/ViewArticle.aspx?ArticleId=18834 pii = 18834. [cited 2010 Aug 7] 10.2807/ese.13.15.18834-en PMC2740836 18445459

[B7] Pappas G Roussos N Falagas ME Toxoplasmosis snapshots: global status of *Toxoplasma gondii *seroprevalence and implications for pregnancy and congenital toxoplasmosis Int J Parasitol 2009 39 1385 1394 10.1016/j.ijpara.2009.04.003 19433092

[B8] Prusa AR Hayde M Gerstl N Pollak A Infection with *Toxoplasma gondii *during pregnancy [in German] Gynäkologische Praxis 2005 29 41 4422265051

[B9] Statistik Austria NUTS-Einheiten 2010 http://www.statistik.at/web_de/statistiken/regionales/regionale_gliederungen/nuts_einheiten/index.html [cited 2010 Aug].

[B10] Nowakowska D Stray-Petersen B Śpiewak E Sobala W Małafiej E Wilczyński J Prevalence and estimated incidence of *Toxoplasma *infection among pregnant women in Poland: a decreasing trend in the younger population Clin Microbiol Infect 2006 12 913 917 10.1111/j.1469-0691.2006.01513.x 16882298

[B11] Griffin J INTCENS: stata module to perform interval-censored survival analysis 2005 http://ideas.repec.org/c/boc/bocode/s453501.html [cited 2010 Aug 7].

[B12] Sensini A *Toxoplasma gondii *infection in pregnancy: opportunities and pitfalls of serological diagnosis Clin Microbiol Infect 2006 12 504 512 10.1111/j.1469-0691.2006.01444.x 16700697

[B13] Statistik Austria Statistiken Bevölkerung 2010 http://www.statistik.at/web_de/statistiken/bevoelkerung/index.html [cited 2010 Aug 7].

[B14] Pass C Inanspruchnahme des Mutter-Kind-Passes. Ein Beispiel für die Wechselwirkung zwischen sozialer Lage und Gesundheit? Schriftenreihe Gesundheitswissenschaften 2001 Linz: Oberösterreichische Gebietskrankenkasse [German: Utilization of the Mother-Child-Booklet. An example for the interaction of social condition and health?]

[B15] Cornu C Bissery A Malbos C Garwig R Cocherel C Ecochard R Factors affecting the adherence to an antenatal screening programme: an experience with toxoplasmosis screening in France Eurosurveill 2009 14 9 http://www.eurosurveillance.org/ViewArticle.aspx?ArticleId=19137 pii = 19137. [cited 2010 Aug 7] 19317970

[B16] Meroni V Genco F Tinelli C Lanzarini P Bollani L Stronati M Spiramycin treatment of *Toxoplasma gondii *infection in pregnant women impairs the production and the avidity maturation of *T. gondii*-specific immunoglobulin G Antibodies Clin Vaccine Immunol 2009 16 1517 1520 10.1128/CVI.00253-09 19692628 PMC2756842

[B17] Giesecke J Modern Infectious Disease Epidemiology 2002 London: Arnold

[B18] Bénard A Salmi LR Mouillet E the European Toxo Prevention Study Group Systematic review on the burden of congenital toxoplasmosis in Europe 2005 http://eurotoxo.isped.u-bordeaux2.fr/[Unpublished report]. Bordeaux (France), [cited 2010 Aug 7]

[B19] Villena I Ancelle T Delmas C Garcia P Brézin AP Thulliez P Toxosurv network and National Reference Centre for Toxoplasmosis Congenital toxoplasmosis in France in 2007: first results from a national surveillance system Eurosurveill 2010 15 25 http://www.eurosurveillance.org/ViewArticle.aspx?ArticleId=19600 pii = 19600. [cited 2010 Aug 7]. 10.2807/ese.15.25.19600-en 20587361

[B20] Hohenauer L Nagl F Vutuc Ch Serologische Untersuchungen zum Mutter Kind Pass. [German: Serological examinations for Mother-Child-Booklet] Mitteilungen der Sanitätsverwaltung 1999 6 8 12

[B21] Hayde M Prusa AR Gratzl R Pollak A Kommentar zur Publikation „Serologische Untersuchungen zum Mutter Kind Pass"von Hohenauer et al Mitteilungen der Sanitätsverwaltung 1999 9 9 12 [German] [Commentary to publication "Serological examinations for Mother-Child-Booklet" by Hohenauer et al.].

[B22] Edelhofer R Prossinger H Infection with *Toxoplasma gondii *during pregnancy: seroepidemiological studies in Austria Zoonoses Public Hlth 2010 57 18 26 10.1111/j.1863-2378.2009.01279.x 19744300

[B23] Hofhuis A van Pelt W van Dunyhoven YTHP Nijhuis CDM Mollema L van der Klis FRM Havelaar AH Kortbeek LM Decreased prevalence and age-specific risk factors for *Toxoplasma gondii *IgG antibodies in The Netherlands between 1995/1996 and 2006/2007 Epidemiol Infect 2011 139 530 538 10.1017/S0950268810001044 20492743

[B24] Cook AJC Gilbert RE Buffolano W Zufferey J Petersen E Jenum PA The European research network on congenital Toxoplasmosis: sources of *toxoplasma *infection in pregnant women: European multicentre case-control-study BMJ 2000 321 142 147 10.1136/bmj.321.7254.142 10894691 PMC27431

[B25] Avelino MM Campos D Jr do Carmo Barbosa de Parada J Pregnancy as a risk factor for acute toxoplasmosis seroconversion Eur J Obstet Gynecol Reprod Biol 2003 108 19 24 10.1016/S0301-2115(02)00353-6 12694964

[B26] Dunn D Wallon M Peyron F Petersen E Peckham C Gilbert R Mother-to-child transmission of toxoplasmosis: risk estimates for clinical counseling Lancet 1999 353 1829 1833 10.1016/S0140-6736(98)08220-8 10359407

[B27] Logar J Šoba B Premru-Sršen T Novak-Antolič Ž Seasonal variations in acute toxoplasmosis in pregnant women in Slovenia Clin Microbiol Infect 2005 11 834 855 10.1111/j.1469-0691.2005.01236.x 16153265

[B28] Sagel U Mikolajczyk R Krämer A Seasonal trends in acute toxoplasmosis in pregnancy in the federal state of upper Austria Clin Microbiol Infect 2010 16 515 517 10.1111/j.1469-0691.2009.02880.x 19622079

